# Fetal surgery for myelomeningocele: initial results of a tertiary public hospital

**DOI:** 10.61622/rbgo/2025rbgo81

**Published:** 2025-10-21

**Authors:** Ingrid Schwach, Gustavo Yano Callado, Sandra Rejane Silva Herbst, Daniel Dante Cardeal, Milton Hikaro Toita, Giselle Darahem Tedesco, Flavia Canhassi Corigliano, Juliana dos Passos Pires, Carolina Leite Drummond, Edward Araujo Júnior

**Affiliations:** 1 Faculdade de Ciências Médicas da Santa Casa de São Paulo Department of Obstetrics and Gynecology São Paulo SP Brazil Department of Obstetrics and Gynecology, Faculdade de Ciências Médicas da Santa Casa de São Paulo, São Paulo, SP, Brazil.; 2 Universidade Federal de São Paulo Escola Paulista de Medicina Department of Obstetrics São Paulo SP Brazil Department of Obstetrics, Escola Paulista de Medicina, Universidade Federal de São Paulo, São Paulo, SP, Brazil.; 3 Hospital Israelita Albert Einstein Faculdade Israelita de Ciências da Saúde Albert Einstein São Paulo SP Brazil Faculdade Israelita de Ciências da Saúde Albert Einstein, Hospital Israelita Albert Einstein, São Paulo, SP, Brazil.; 4 Universidade de São Paulo Department of Obstetrics and Gynecology São Paulo SP Brazil Department of Obstetrics and Gynecology, Universidade de São Paulo, São Paulo, SP, Brazil.; 5 Universidade Municipal de São Caetano do Sul Discipline of Woman Health São Caetano do Sul SP Brazil Discipline of Woman Health, Universidade Municipal de São Caetano do Sul, São Caetano do Sul, SP, Brazil.

**Keywords:** Myelomeningocele, Fetus, Perinatal outcomes, Treatment outcomes

## Abstract

**Objective:**

To describe preliminary results of open fetal myelomeningocele surgeries performed at a tertiary public hospital in São Paulo, Brazil, analyzing epidemiological aspects as well as maternal and fetal perioperative complications.

**Methods:**

This retrospective cohort study included 25 pregnant women whose fetuses were diagnosed with myelomeningocele and underwent open intrauterine surgery between February 2019 and October 2023. Maternal demographic data, surgical outcomes, and neonatal variables were collected from medical records. Statistical analyses included Chi-squared test, Fisher's exact test, Mann–Whitney U test, and Pearson's correlation coefficient (r).

**Results:**

The mean gestational age (GA) at surgery was 25.7 ± 1.2 weeks, and the mean GA at delivery was 34.3 ± 2.0 weeks. Preterm birth was the most common complication (40%), followed by premature rupture of ovular membranes (32%). Two stillbirths occurred (8%) at 31 and 33 weeks of gestation. Median Apgar scores at 1 and 5 minutes were 8 and 9, respectively. Low birth weight (<2,500 g) was observed in 44% of neonates. Neonatal hospitalization was significantly longer in cases of preterm birth (p = 0.044), and intensive care unit stay was longer in deliveries before 34 weeks (p = 0.044). No correlation was found between GA at intrauterine surgery and GA at delivery (r = 0.252, p = 0.22).

**Conclusion:**

Fetal myelomeningocele surgery was successfully performed in a tertiary public hospital. However, preterm birth remains a major concern, as consistently reported in the literature. Nevertheless, the results confirm that intrauterine repair of myelomeningocele is indeed feasible in the public healthcare setting.

## Introduction

Intrauterine myelomeningocele repair is a major advance in the management of some forms of spina bifida, reducing neurological complications and improving outcomes. The ‘*Management of Myelomeningocele Study’* (MOMS) demonstrated that prenatal surgery lowers the rates of hindbrain herniation, reducing ventricular shunt dependence by the age of 12 months, and also enhancing motor function at 30 months old.^(1-3)^ Subsequent studies supported fetal repair due to ambulatory benefits status and hydrocephalus prevention.^(4)^

Nowadays, long-term data indicates better mobility, lower incidence of Chiari Type II, hydrocephalus, and, as expected, fewer shunt-related surgeries in children undergoing prenatal repair. Some aspects still lack validation, such as improvement on cognition.^(5)^

Despite all positive aspects, the procedure carries maternal and fetal risks, as follows: preterm birth (delivery <37 weeks), oligohydramnios, chorioamnionitis, placental abruption, uterine dehiscence, pulmonary edema, and others.^(1,6)^ For this reason, it is well established that fetal surgery indication requires a careful balance between risks and benefits, within a multidisciplinary team.

Data from the past two decades indicate that intrauterine repair significantly improves outcomes in individuals who, when treated postnatally, are frequently subject to lifelong disabilities. This is equal to say that expanding access of such therapy in public healthcare systems in low- and middle-income countries (LMICs), where the prevalence of spina bifida is higher (possibly due to limited prenatal care and folic acid supplementation), represents more than a personal benefit, but a social and governmental gain, implicating in less lifelong complications, reduction of long-term health costs and improvement of life quality for those affected.^(7)^

The aim of this study is to report our experience with the first 25 cases of fetal myelomeningocele repair in a Brazilian tertiary public hospital in São Paulo, and presents epidemiological data, as well as maternal and fetal complications associated with the procedure.

## Methods

This is a retrospective cohort study, conducted with a sample of 25 pregnant women whose fetuses were diagnosed with myelomeningocele, and underwent intrauterine surgical repair at the Santa Casa de Misericórdia de São Paulo, Brazil, between February 2019 and October 2023.

All inclusion criteria mirrored the MOMS trial,^(1)^ except that the upper GA limit was set at 27 + 6 weeks and maternal BMI ≤40 kg/m², as determined by our institutional fetal-surgery board.

Data collection was performed by reviewing medical records, available at hospital database, followed by a systematic analysis of the extracted content. Patients who discontinued follow-up care at the institution were excluded.

Demographic characteristics, including marital status, educational degree, number of pregnancies, maternal and gestational age (GA) at intrauterine surgery, chronic diseases, peri-conception folic acid intake, maternal body mass index (BMI), maternal addictions and allergies, were analyzed.

Surgical outcomes included complications, such as desaturation, need of intraoperative blood transfusion, chorioamnionitis, oligohydramnios, premature rupture of ovular membranes (PROM), scar dehiscence, chorioamniotic separation, uterine rupture, placental abruption, preterm birth, need of blood transfusion at delivery, uterine atony, need of hysterectomy, maternal death, and stillbirth.

**Figure 1:**
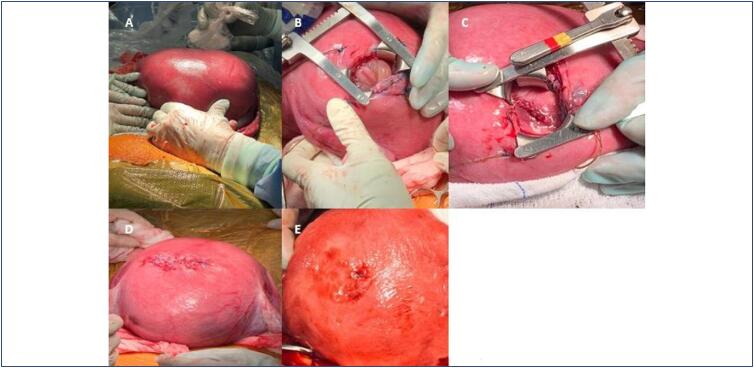
(A) Exteriorization of the uterus with ultrasound mapping of placental insertion and identification of fetal position. (B) Exposure of the fetal myelomeningocele by hysterotomy with an Ankeney retractor. (C) Repair of the myelomeningocele by the neurosurgical team. (D) Hysterorrhaphy with identification of the suture in the uterine serosa. (E) Identification of the uterine scar from myelomeningocele repair after cesarean section

The surgical technique applied was adapted from the MOMS study protocol.^(1)^ The intrauterine surgery of myelomeningocele was performed under combined epidural and general anesthesia, with the patient in the dorsal decubitus position, urethral Foley catheter placed, and pneumatic compression devices applied to the lower limbs. Following asepsis and antisepsis, sterile draping was completed. An enlarged Pfannenstiel incision was carried out to allow uterine exteriorization. Fetal positioning was determined via ultrasonography, ensuring that the opening site was away from the placental edge, umbilical cord, and as close as possible to the lesion. Two anchoring sutures were placed transfixing the myometrium under ultrasound guidance at the superior and inferior edges of the marked site using Vicryl 0.0. A longitudinal hysterotomy of 3.5 to 4.0 cm was then performed, depending on lesion size. The amniotic membrane was identified, opened, and the amniotic fluid was partially aspirated, with temporary hemostatic clamping. The amniotic membrane was then sutured to the underlying myometrium with a purse-string suture using Vicryl 2.0. The fetal dorsum was exposed using an Ankenney retractor, allowing the neurosurgical team to dissect the herniated sac and repair the neural defect. Once the correction was completed, the uterus was closed, and approximately 300 mL of warm 0.9% saline solution was infused into the amniotic cavity to restore fluid volume. The closure was performed in multiple layers: interrupted "X" sutures with Vicryl 0.0 for the myometrium and amniotic membrane, two U-shaped sutures with Ethibond 5.0 for the myometrium and serosa, followed by a continuous serosal closure with Vicryl 2.0. The uterus was then repositioned into the pelvic cavity. No antibiotic was added to the saline solution infused into the amniotic cavity. However, a single vial of vancomycin was applied locally into the uterine cavity during closure, in accordance with the institutional infection prevention protocol. Hemostasis was carefully reviewed before closure of the abdominal layers. Fetal well-being was continuously monitored via ultrasound throughout the procedure (Figure 1).

Postoperatively, all patients received intravenous magnesium sulfate for 24 hours. After a 4-hour interval, oral nifedipine (20 mg every 6–8 hours) and vaginal progesterone (200 mg daily) were introduced when clinically indicated to suppress uterine contractions.

The demographic data were presented as mean ± standard deviation (SD), along with the median and the range (minimum–maximum) for quantitative variables. For categorical variables, data were shown as absolute numbers and percentages n (%). Statistical analyses were performed using Chi-squared or Fisher's exact test for categorical variables. Additionally, Mann-Whitney U tests were used to compare continuous variables such as hospitalization duration. Pearson's correlation coefficient (r) was applied to evaluate the relationship between gestational age at intrauterine surgery and gestational age at delivery.

To assess fetal growth, birth weights were plotted against the Intergrowth-21st standards for unsexed newborns, according to gestational age at delivery.^(8)^ Gestational age was converted into decimal weeks, and percentiles were calculated based on standard reference values. Neonates were then classified as small for gestational age (SGA, <10th percentile), appropriate for gestational age (AGA, 10th–90th percentile), or large for gestational age (LGA, >90th percentile).

A significance level of p<0.05 was considered statistically significant. Analyses were conducted in R software version 4.3.3.

Ethical approval for this study was obtained from the Ethics Committee of Faculdade de Ciências Médicas da Santa Casa de São Paulo (FCMSCSP) number 6.469.626 (Certificado de Apresentação de Apreciação Ética – CAAE: 69234523.0.0000.5479), ensuring compliance with ethical standards for research involving human subjects.

## Results

A total of 25 fetal surgeries were performed for prenatal repair of myelomeningocele, between February 2019 and October 2023. The mean maternal age at intrauterine surgery was 25.4 ± 1.2 years. The mean GA at intrauterine surgery was 25.7 ± 1.2 weeks. Regarding marital status, 8(32%) women were married, 10(40%) were single, 6(24%) were in cohabiting relationships, and 1(4%) did not provide information. Among the 25 operated cases, 7 were classified as myeloschisis and 18 as myelomeningocele. The upper limit of the lesion ranged from L1 to S1. Planned pregnancies accounted for 3(12%) of cases, while 11(44%) were unplanned, and 11(44%) had missing information. Regarding parity, 11(44%) were in their first pregnancy, 6(24%) in their second, and 8(32%) had three or more pregnancies. Obstetric histories revealed that 6(24%) had at least one previous vaginal delivery, 8(32%) had undergone a cesarean section, and 4(16%) reported a history of at least one abortion. For educational levels, 1(4%) had primary education, 8(32%) secondary education, 4 (16%) university education, and 1(4%) postgraduate education. Education status was not informed by 11(44%). Chronic diseases were reported in 9(36%) cases. Regarding folic acid use, 6(24%) used it before pregnancy, 9(36%) began after becoming pregnant, 5(20%) did not use it at all, and 5(20%) did not disclose their status. Lastly, 1(4%) reported a past of drug addictions, and 4(16%) had allergies (Table 1).

**Table 1:** Maternal demographic characteristics

Characteristics		n(%)
Maternal age at surgery (years)		25.4 ± 1.2 (25.4; 22.3–27.6)
Gestational age at surgery (weeks)		25.7 ± 1.2 (26.0; 23.3–27.4)
Marital status		
	Married	8(32)
	Single	10(40)
	Cohabitation	6(24)
	Not informed	1(4)
Planned gestation		
	Yes	3(12)
	No	11(44)
	Not informed	11(44)
Number of pregnancies		
	1	11(44)
	2	6(24)
	≥3	8(32)
Obstetric history		n(%)
	≥1 previous vaginal delivery	6(24)
	≥1 previous cesarean section	8(32)
	≥1 previous abortion	4(16)
Highest education level		
	Primary education	1(4)
	Secondary education	8(32)
	University education	4(16)
	Postgraduate education	1(4)
	Not informed	11(44)
Chronic diseases		9(36)
Folic acid use		
	Yes, before getting pregnant	6(24)
	Yes, started after getting pregnant	9(36)
	No	5(20)
	Not informed	5(20)
Addictions		1(4)
Allergies		4(16)

Quantitative data are reported: mean ± standard deviation (median; minimum value–maximum value)

In examining maternal, fetal, intraoperative, and postoperative complications, we present the following findings (Table 2): three cases of maternal desaturation (12%), four instances of intraoperative blood transfusion (16%), one case of chorioamnionitis documented at delivery (4%), five patients who developed oligohydramnios (20%), and eight patients who experienced PROM (32%). Scar dehiscence was observed in two patients (8%), while chorioamniotic separation occurred in one case (4%). Notably, there were no incidents of uterine rupture (0%), and one case of placental abruption was reported eight weeks post-fetal surgery but did not result in fetal death (4%). Preterm birth emerged as the most prevalent complication in our cohort, affecting ten patients (40%). At the time of delivery, no patients required blood transfusions (0%). Immediate postpartum uterine atony was documented in one case (4%), which was successfully managed using both pharmacological and mechanical interventions. None of the patients required a hysterectomy, and there were no reported maternal fatalities (0%). However, stillbirth occurred in two cases (8%): one at 31 weeks of gestation (following PROM) and another at 33 weeks, with no significant obstetric events noted. Among the pregnant patients, 20% had no complications, 24% had one, 48% had two, 4% had three, and 4% had five. The latter case involved blood transfusion, postoperative oxygen therapy, PROM at 31 weeks, chorioamnionitis, and preterm birth, along with reported domestic violence after hospital discharge. The mean postoperative hospital stay was 5.4 ± 1.4 days. The mean length of the intensive care unit (ICU) was 2.2 ± 0.8 days, while the mean ward stay was 3.2 ± 1.5 days. The mean gestational age at delivery was 34.3 ± 2 weeks. The mean maternal hospital stay after delivery was 3.4 ± 0.8 days. Apgar scores at 1 and 5 minutes were recorded for all neonates except for the two stillbirths. The median Apgar score was 8 and 9 for 1 and 5 minutes respectively. Most neonates (19/25, 76%) had Apgar scores ≥7 at both time points, reflecting an overall good status in the immediate postnatal adaptation period. However, three newborns (12%) had Apgar scores below 7 at 1 minute, and one (4%) remained below 7 at 5 minutes. Birth weights ranged from 1,350 g to 3,245 g, with a mean birth weight of 2,275 ± 503 g. Of the 23 live births, 11 (44%) neonates had birth below 2,500 g, classifying them as low weight at birth, while three (12%) were below 1,750 g, meeting the criteria for very low birth weight. The highest birth weight was 3,245 g, while the lowest was 1,350 g.

**Table 2:** Perioperative and postoperative complications

Complication		n(%)
Desaturation		3(12)
Need for blood transfusion during surgery		4(16)
Chorioamnionitis		1(4)
Oligohydramnios		5(20)
PROM		8(32)
Scar dehiscence		2(8)
Chorioamniotic separation		1(4)
Uterine rupture		0(0)
Placental abruption		1(4)
Preterm birth		10(40)
Need for blood transfusion during delivery		0(0)
Uterine atony		1(4)
Need for hysterectomy		0(0)
Maternal death		0(0)
Stillbirth		2(8)
Number of complications		
	0	5(20)
	1	6(24)
	2	12(48)
	3	1(4)
	5	1(4)
Postoperative hospital stay (days)		5.4 ± 1.4 (5; 4–11)
ICU stay duration (days)		2.2 ± 0.8 (2; 1–5)
Ward stay duration (days)		3.2 ± 1.5 (3; 2–9)
GA at delivery (weeks)		34.3 ± 2 (34.6; 30.1–37.3)
Maternal hospital stay after delivery (days)		3.4 ± 0.8 (3; 3–7)

Quantitative data are reported: mean ± standard deviation (median; minimum value–maximum value). PROM - premature rupture of ovular membranes; ICU - intensive care unit; GA - gestational age

No significant association was identified between maternal demographic characteristics and perinatal complications. The analysis was conducted in two ways: individually for each characteristic and by combining all adverse outcomes into a single variable. However, results from the Mann-Whitney U test revealed that the length of postpartum hospitalization was significantly longer for patients who experienced preterm birth (p = 0.044). Similarly, the duration of neonatal ICU stay was significantly extended for cases where delivery occurred before 34 weeks of gestation (p = 0.044) (Table 3).

**Table 3:** Preterm birth and gestational age at delivery.

	Premature birth					GA at delivery				
		n	Mean Rank	Sum of Ranks	p-value^*^		n	Mean Rank	Sum of Ranks	p-value^*^
GA at surgery	No	15	14.8	221.5	0.141	<34 weeks	10	12.3	122.5	0.677
	Yes	10	10.4	103.5		≥34 weeks	15	13.5	202.5	
	Total	25				Total	25			
ICU stay	No	15	13.2	198.5	0.82	<34 weeks	10	16.1	161	0.044
	Yes	10	12.7	126.5		≥34 weeks	15	10.9	164	
	Total	25				Total	25			
Ward stay	No	15	13	195	1	<34 weeks	10	13	130	1
	Yes	10	13	130		≥34 weeks	15	13	195	
	Total	25				Total	25			
Maternal hospital stay after delivery	No	15	11.1	166.5	0.044	<34 weeks	10	14.7	146.5	0.243
	Yes	10	15.9	158.5		≥34 weeks	15	11.9	178.5	
	Total	25				Total	25			
Maternal age	No	15	12.6	189	0.738	<34 weeks	10	12.1	120.5	0.597
	Yes	10	13.6	136		≥34 weeks	15	13.6	204.5	
	Total	25				Total	25			
Total of pregnancies	No	15	13.4	201	0.724	<34 weeks	10	10	100	0.077
	Yes	10	12.4	124		≥34 weeks	15	15	225	
	Total	25				Total	25			
Previous vaginal delivery	No	15	13.4	201.5	0.63	<34 weeks	10	11.1	110.5	0.148
	Yes	10	12.4	123.5		≥34 weeks	15	14.3	214.5	
	Total	25				Total	25			
Previous cesarean section	No	15	13.8	207	0.414	<34 weeks	10	12.6	126	0.785
	Yes	10	11.8	118		≥34 weeks	15	13.3	199	
	Total	25				Total	25			
Previous abortion	No	15	11.8	177.5	0.127	<34 weeks	10	12.3	122.5	0.513
	Yes	10	14.8	147.5		≥34 weeks	15	13.5	202.5	
	Total	25				Total	25			
BMI	No	12	10.5	126	0.67	<34 weeks	9	12.3	111	0.394
	Yes	9	11.7	105		≥34 weeks	12	10	120	
	Total	21				Total	21			

* Mann-Whitney U test. BMI - body mass index; GA - gestational age; ICU - intensive care unit

As expected, higher gestational age at delivery was associated with a lower rate of maternal and fetal complications (p = 0.008). No association was found between gestational age at delivery and maternal age (Table 4). Additionally, complications (maternal or fetal) were not linked to the length of stay in the ICU or ward, the number of pregnancies, deliveries, or body mass index (BMI). Pearson's correlation analysis indicated no significant relationship between gestational age at surgery and gestational age at birth (r = 0.252, p = 0.22).

**Table 4:** Comparison between maternal and surgical characteristics and complication

Variable	Complication	n	Mean	SD	p-value^*^
GA (days) at surgery	Yes	20	178.2	9.4	0.795
	No	5	177.0	7.7	
GA (days) at delivery	Yes	20	236.6	13.3	0.008
	No	5	254.8	7.5	
Maternal age (years)	Yes	20	25.5	5.3	0.235
	No	5	28.8	6.4	

* Student t-test. GA - gestational age; SD - standard deviation

Elective cesarean sections were scheduled for five patients (20%), all of whom delivered at 37.0 weeks of gestation. Among the remaining patients, eight (32%) experienced PROM, followed by spontaneous labor and subsequent preterm delivery. In two cases (8%), delivery was indicated due to ultrasound signs of scar dehiscence, characterized by uterine wall thickness less than 3 mm, even in the absence of uterine contractions. One case (4%) involved preterm labor with rapid progression, associated with maternal pain and uterine hypertonia, and was found to have cervical dilation of 4.0 cm at the time of cesarean delivery; placental abruption was diagnosed intraoperatively at 36.0 weeks. Overall, 10 cases (40%) presented with preterm labor: in five of them, delivery was indicated due to associated oligohydramnios, while in the remaining five, delivery occurred due to failed tocolysis. Birth weights were assessed using Intergrowth-21st standards. Two neonates (8.7%) were classified as small for gestational age (SGA), 15 (65.2%) as appropriate for gestational age (AGA), and 6 (26.1%) as large for gestational age (LGA), suggesting that most low-weight cases were due to prematurity rather than fetal growth restriction. A preliminary analysis of postnatal outcomes showed a cerebrospinal fluid shunt rate of 30% (7/23), although a full evaluation of neurological outcomes lies beyond the scope of the present study.

## Discussion

This study presents preliminary results on a series of 25 fetal myelomeningocele surgery in a tertiary public hospital in São Paulo, Brazil, and reports both epidemiological data and perioperative complications. Despite limitations found in our public health system, our results confirm that fetal surgery is feasible in a Brazilian tertiary public center, though challenges such as prematurity (40%) and PROM (32%), remain significant concerns, in accordance with what is found in existing literature, where prematurity is one of the main risks associated with fetal myelomeningocele surgery.^(9)^

In our cohort, all patients were admitted to the ICU for at least 24 hours following fetal surgery. This decision was based on institutional protocol, which includes routine administration of intravenous magnesium sulfate during the postoperative period to reduce uterine contractility and lower the risk of preterm labor. Therefore, ICU admission was not necessarily a response to postoperative complications, but rather a preventive measure related to pharmacologic management. Although ICU use may increase the overall cost of care, its standardized application in our setting reflects a cautious approach aimed at optimizing maternal outcomes and ensuring patient safety during the immediate recovery phase.

In Brazil, the first set of fetal surgeries for myelomeningocele in a public hospital was held in another tertiary center in São Paulo and presented 39 cases recruited between 2015 and 2019. Like in ours, the main complications found on that series were PROM and preterm birth with rates as high as 46.2% and 51.3% respectively.^(10)^ The lower complication rate observed in our study is probably a consequence of technical improvements acquired since MOMS trial has been concluded.

Neonatal outcomes in our population were generally favorable, (median 1-minute Apgar score equal to 8 and a 5-minute score was 9), indicating good response during the postnatal adaptative period, and no impact of the surgery on this particular aspect. Additionally, nearly half of the neonates (44%) were classified as low birth weight (<2,500 g), which is consistent with prematurity-related growth restriction.^(11)^ When comparing our data to the initial experiences reported by two centers in Chile, we noted that among their 58 fetuses who underwent intrauterine surgery between 2011 and 2019, there were three intrauterine deaths (5.1%), with a mean birth weight of 2,172 ± 751 g (median 2,212; range, 850–3,980).^(12)^ This information indicates a comparable mean birth weight between the two populations, with our cohort showing a mean birth weight of 2,275 ± 503 g and a median of 2,250 g. Additionally, our data reflects a slightly lower rate of intrauterine deaths.

Statistical analysis revealed no significant associations between maternal demographic characteristics and perinatal complications, suggesting that factors such as education level, periconceptional folic acid intake, and marital status did not influence surgical or neonatal outcomes in this cohort. However, postpartum hospitalization was significantly longer in cases of preterm birth (p = 0.044), as well as ICU stay in neonates born before 34 weeks (p = 0.044). These findings reinforce the importance of developing strategies that could lead to optimized perinatal management, reducing the impact of preterm birth on both maternal and neonatal morbidity.

Pulmonary edema, a common maternal complication following surgery, was also observed in the largest Brazilian cohort study conducted at a private institution in São Paulo. In their sample, which included 237 fetal surgeries performed between 2011 and 2017, 2.5% of the population developed pulmonary edema, and 2.1% required blood transfusions at delivery.^(13)^ While this finding highlights a notable aspect of their study, the absence of such complications in our population may be attributed to the small size of our sample.

Our results align with those from the MOMS trial, indicating that fetal myelomeningocele surgery enhances neurological outcomes while simultaneously increasing maternal and obstetric risks, particularly concerning prematurity and PROM.^(1,14)^ The mean gestational age at delivery in our study was 34.3 weeks, which is comparable to the 34.1 weeks reported in the MOMS trial. However, our PROM rate of 32% was slightly lower than the 46% recorded in the MOMS trial.^(1)^ Despite the small sample size, the reduction in the size of the uterine incision likely contributed to this favorable outcome. In contrast, a reference center in Argentina that analyzed 21 fetuses who underwent intrauterine surgery between 2015 and 2017 reported a PROM rate of 52%, which is higher than our finding, along with a mean gestational age at delivery of 34.2 weeks, which is similar to the results of our study.^(15)^

Although the MOMS trial limited surgery to before 26 weeks,^(1)^ our protocol allowed procedures up to 27 + 6 weeks after multidisciplinary evaluation. This adjustment reflects real-world challenges in the public health system, such as delayed diagnosis and referral. In our cohort, three patients underwent surgery beyond 26 weeks, with no increase in adverse maternal or perinatal outcomes. While surgery at later gestational ages may pose theoretical risks due to decreased uterine compliance, our findings suggest that selected cases can safely benefit from intervention beyond the original MOMS criteria.

The Apgar scores observed in our cohort were comparable to those reported in other fetal surgery programs, where the majority of neonates achieved Apgar scores of ≥7 at five minutes, indicating satisfactory neonatal stability despite prematurity.^(16)^ The high incidence of low birth weight (44%) likely correlates with the observed rates of prematurity. A standardized postoperative protocol including magnesium sulfate, followed by nifedipine and progesterone, was applied to prevent preterm labor. This approach may have contributed to the lower PROM.

Notably, GA at intrauterine surgery was not correlated with GA at delivery (r=0.252, p=0.22), suggesting that surgical timing alone does not predict pregnancy duration. This is in accordance with previous studies, indicating that other factors, such as membrane integrity, uterine contractility, and maternal characteristics, might play a more important role in determining pregnancy length after fetal surgery.^(17)^

The findings mentioned above emphasize the importance of specialized perioperative management in order to mitigate prematurity-related complications. Given that both postpartum in hospital and ICU stay were significantly prolonged in preterm births, targeted interventions such as enhanced tocolysis, maternal corticosteroid administration, and close post-surgical monitoring protocols certainly help reducing the burden of prematurity.^(18,19)^ Cools et al.^(20)^ compared 24 prenatal and 34 postnatal meningomyelocele repairs and observed that prenatal repair fetuses were delivered more prematurely and with lower birth weights, although the neonatal ICU permanence was similar between the two groups. Furthermore, infants with prenatal repair had fewer hospital readmissions at 30 days, 60 days, and 1 year than the postnatal repair group.

Expanding access to fetal myelomeningocele repair in LMICs is a critical goal, particularly given the high prevalence of neural tube defects in these regions, often due to inadequate folic acid supplementation and limited prenatal and postnatal care for affected individuals.^(15,21)^ The successful establishment of a fetal surgery program in a public hospital demonstrates that such procedures can be integrated into resource-limited healthcare systems, provided that adequate infrastructure and multidisciplinary expertise are available. This integration can lead to a reduction in lifelong complications and, consequently, lower governmental costs associated with these conditions.

Lastly, the limitations of this study must be acknowledged. Both the retrospective design and the small sample size (n=25) pose constraints on our findings. Additionally, long-term neonatal follow-up data are not yet available, preventing an assessment of the procedure's full impact on motor function, ambulatory status, and shunt dependency. Finally, this study did not aim to evaluate postnatal neurological outcomes, such as shunt dependency or motor function, as its primary focus was on maternal and perinatal variables. Future studies should focus on longitudinal follow-up of these neonates to evaluate neurodevelopmental outcomes and quality of life.

## Conclusion

Fetal myelomeningocele surgery was successfully carried out, demonstrating the feasibility of the procedure in a public healthcare center, performed by a multidisciplinary and well-trained team. The complication rates associated with the surgery were consistent with those reported in the literature, particularly regarding preterm birth, PROM, and neonatal low birth weight. Although neonatal outcomes were generally favorable, preterm birth remains a significant concern, underscoring the need for improved management strategies in these cases. We recognize that expanding access to fetal surgery in developing countries presents an opportunity to reduce the burden of disabilities and enhance the quality of life for affected individuals and their families. Nevertheless, further research is necessary to evaluate long-term neurological outcomes.

## References

[1] Adzick NS, Thom EA, Spong CY, Brock JW, Burrows PK, Johnson MP (2011). A randomized trial of prenatal versus postnatal repair of myelomeningocele. N Engl J Med.

[2] Houtrow AJ, Thom EA, Fletcher JM, Rivera M, Flynn L, Burrows PK (2020). Prenatal repair of myelomeningocele and school-age functional outcomes. Pediatrics.

[3] Farmer DL, Thom EA, Brock JW, Burrows PK, Johnson MP, Howell LJ (2018). The management of myelomeningocele study: full cohort 30-month pediatric outcomes. Am J Obstet Gynecol.

[4] Bauer DF, Beier AD, Nikas DC, Assassi N, Blount J, Durham SR (2019). Congress of neurological surgeons systematic review and evidence-based guideline on the management of patients with myelomeningocele: whether prenatal or postnatal closure affects future ambulatory status. Neurosurgery.

[5] Kohútková M, Horn F (2024). Arnold-Chiari malformations in pediatric patients after fetal surgery for meningomyelocele. J Clin Med.

[6] Koch VH, Lopes M, Furusawa E, Vaz K, Barroso U (2024). Multidisciplinary management of people with spina bifida across the lifespan. Pediatr Nephrol.

[7] Joyeux L, Danzer E, Flake AW, Deprest J (2018). Fetal surgery for spina bifida aperta. Arch Dis Child Fetal Neonatal Ed.

[8] Villar J, Cheikh Ismail L, Victora CG, Ohuma EO, Bertino E, Altman DG (2014). International standards for newborn weight, length, and head circumference by gestational age and sex: the Newborn Cross-Sectional Study of the INTERGROWTH-21st Project. Lancet.

[9] Johnson MP, Bennett KA, Rand L, Burrows PK, Thom EA, Howell LJ (2016). The Management of Myelomeningocele Study: obstetrical outcomes and risk factors for obstetrical complications following prenatal surgery. Am J Obstet Gynecol.

[10] Rocha LS, Bunduki V, Amorim AG, Cardeal DD, Matushita H, Fernandes HS (2021). Open fetal myelomeningocele repair at a university hospital: surgery and pregnancy outcomes. Arch Gynecol Obstet.

[11] Katz J, Lee AC, Kozuki N, Lawn JE, Cousens S, Blencowe H (2013). Mortality risk in preterm and small-for-gestational-age infants in low-income and middle-income countries: a pooled country analysis. Lancet.

[12] Sepulveda W, Corral E, Alcalde JL, Otayza F, Müller JM, Ravera F (2020). Prenatal repair of spina bifida: a 2-center experience with open intrauterine neurosurgery in Chile. Fetal Diagn Ther.

[13] Moron AF, Barbosa MM, Milani H, Sarmento SG, Santana E, Suriano IC (2018). Perinatal outcomes after open fetal surgery for myelomeningocele repair: a retrospective cohort study. BJOG.

[14] Moldenhauer JS, Adzick NS (2017). Fetal surgery for myelomeningocele: after the Management of Myelomeningocele Study (MOMS). Semin Fetal Neonatal Med.

[15] Etchegaray A, Palma F, De Rosa R, Russo RD, Beruti E, Fregonese R (2018). [Fetal surgery for myelomeningocele: obstetric evolution and short-term perinatal outcomes of a cohort of 21 cases]. Surg Neurol Int.

[16] Sanz Cortes M, Chmait RH, Lapa DA, Belfort MA, Carreras E, Miller JL (2021). Experience of 300 cases of prenatal fetoscopic open spina bifida repair: report of the International Fetoscopic Neural Tube Defect Repair Consortium. Am J Obstet Gynecol.

[17] Kahr MK, Winder F, Vonzun L, Meuli M, Mazzone L, Moehrlen U (2020). Risk factors for preterm birth following open fetal myelomeningocele repair: results from a prospective cohort. Fetal Diagn Ther.

[18] Novoa Y Novoa V, Shazly S, Araujo E, Tonni G, Ruano R (2020). Tocolysis for open prenatal repair of myelomeningocele: systematic review. J Matern Fetal Neonatal Med.

[19] Bennett KA, Carroll MA, Shannon CN, Braun SA, Dabrowiak ME, Crum AK (2014). Reducing perinatal complications and preterm delivery for patients undergoing in utero closure of fetal myelomeningocele: further modifications to the multidisciplinary surgical technique. J Neurosurg Pediatr.

[20] Cools M, Northam W, Goodnight W, Mulvaney G, Elton S, Quinsey C (2019). Thirty-day medical and surgical readmission following prenatal versus postnatal myelomeningocele repair. Neurosurg Focus.

[21] Rocha LS, Bunduki V, Cardeal DD, Amorim AG, Nani FS, Peres SV (2023). Risk factors for shunting at 12 months following open fetal repair of spina bifida by mini-hysterotomy. J Perinat Med.

